# Validation of an alternate method for monitoring the presence of cows at the feed bunk in a Calan Broadbent Feeding System using a 3-axis, data-logging accelerometer

**DOI:** 10.3168/jdsc.2021-0117

**Published:** 2021-10-22

**Authors:** Cesar Matamoros, Rebecca A. Bomberger, Kevin J. Harvatine

**Affiliations:** Department of Animal Science, Penn State University, University Park 16802.

## Abstract

Daily dry matter intake is a key observation in dairy nutrition, and observation of feeding behavior provides insight into the physiological control of hunger and satiety that regulate intake. The objective of the study was to develop and validate an alternative method to observe feeding behavior, including meal length and frequency, in a Calan Broadbent Feeding System (American Calan) using a 3-axis accelerometer (Hobo Pendant G, Onset Computer Corp.). Sensors were mounted between the door and the feed divider using commonly available materials without making permanent modifications to the feeding system. Forty-eight sensors were deployed with a recording frequency of 30 s for the last 7 d of each period in a crossover experiment with 24 multiparous and 24 primiparous animals housed in a freestall barn. The tilt angle on the Z-axis was used to determine when the door was open to indicate feeding activity. The sensor system was in very high agreement with 6 h of visual observation (Cohen’s κ = 0.92 ± 0.014; estimate ± 95% confidence interval). The minimum intermeal interval is the time between 2 feeding bouts that is still considered one meal. This essential criterion to characterize meals was calculated by determining the intersection of a mixture of Gaussian distributions fitted to the log-transformed between-feeding intervals. The best fitting mixture of Gaussian distributions was determined with the distribution module of JMP Pro 14.3.0 (SAS Institute Inc.). The minimum intermeal interval was 31.3 min using the best fitting model, a mixture of 3 Gaussian distributions. Using the determined minimum intermeal interval, meal length averaged 37.3 min/meal and meal frequency averaged 7.3 meals/d. In conclusion, data-logging 3-axis accelerometers are adequate to monitor presence of cows in the feed gate in the Calan Broadbent Feeding System, and this approach allows for reasonable estimation of meal length and frequency.

## Summary

A method was developed using a 3-axis accelerometer to detect cow presence at the bunk in a Calan Broadbent feeding system to characterize feeding behavior. The sensor was at 0° when the door was closed and near 60° when the door was open, allowing clear distinction of the presence of a cow at the feed bunk, in very high agreement with visual observations. In conclusion, accelerometers provided robust monitoring of the presence of cows in the feed gate in the Calan Broadbent Feeding System, and this approach allows for economical and reasonable observation of feeding behavior.

## Graphical Abstract

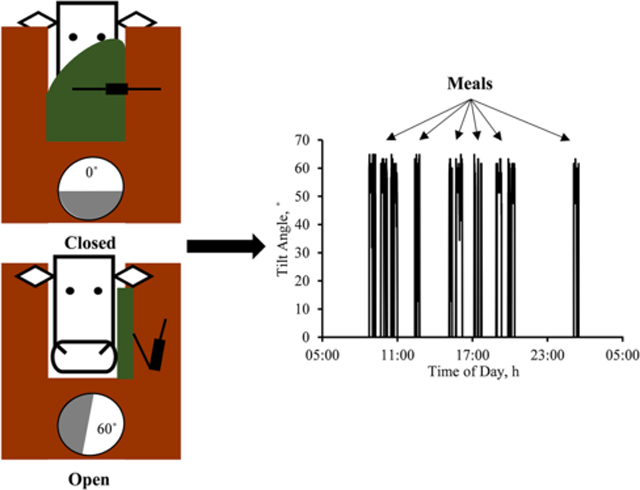

Daily DMI is a key observation in dairy nutrition and is a function of the number and size of meals that is the result of hunger and satiety signals that vary across the day (Allen, 2014). Monitoring feeding behavior provides an understanding of the physiological controls of feed intake in dairy cattle (Allen, 2014). Furthermore, the number of meals per day has been shown to be positively associated with milk fat yield and tends to be associated with DMI and milk production in lactating dairy cows (Johnston and DeVries, 2018). The timing of feed intake across the day is also important in the entrainment of central and peripheral circadian rhythms in lactating dairy cows, including regulation of the mammary gland (Niu et al., 2014; Salfer and Harvatine, 2020). The Calan Broadbent Feeding System (American Calan) is commonly used to observe individual DMI in group-housed cows, but it does not allow observation of feeding behavior without modification by installation of change-of-state sensors or labor-intensive visual observation methods (Vasilatos and Wangsness, 1980; Krawczel et al., 2012).

Feeding behavior observation systems are available for group-housed animals, but they vary in complexity and cost. Time-lapse video photography has been validated to monitor feeding behavior in lactating dairy cows (Vasilatos and Wangsness, 1980; Overton et al., 2002) but requires considerable time and labor for data processing. Automated commercial monitoring systems, such as the GrowSafe (GrowSafe Systems; Mendes et al., 2011) or RIC-2Discover (Hokofarm Group; Chapinal et al., 2007), are available, and custom systems can be built (Bach et al., 2004) but require a substantial investment.

Krawczel et al. (2012) utilized a change-of-state sensor to monitor door position in Calan Broadbent doors to observe feeding behavior. Based on this principle, we report an alternative method using a 3-axis accelerometer data logger to measure the degree of tilt of the sensors as a proxy of when the cow is in the Calan gate (i.e., feeding door is open) and subsequently estimate meal characteristics such as frequency and length. The first objective of the study was to develop and validate an alternative method to observe feeding behavior in a Calan Broadbent Feeding System based on the movement of the feeding door using the HOBO Pendant G data logger (Onset Computer Corp.). These accelerometers are commonly used to measure lying time and behavior in cows (Ledgerwood et al., 2010) and calves (Costa et al., 2021), thus providing multiple applications after their purchase. The second objective was to determine the minimum intermeal interval, also known as meal criterion. The minimum intermeal interval is used to define when 2 feeding events are considered to be a single meal or 2 separate meals.

A HOBO Pendant G accelerometer was hung between the feeding door and the divider between feed bunks. Specifically, a sensor bracket was made from approximately 8-cm-long pieces of 5.1-cm-diameter schedule 40 polyvinyl chloride (PVC) construction pipe. The pipe was cut into 3 pieces through the transverse plane (Figure 1A). A hole was drilled at each end for attachment to the door and feed divider and in the middle to attach the sensor. The sensor was attached to the PVC pipe piece with nylon cable ties and an elastic band was placed at one end (Figure 1A). A 25.4 mm × 25.4 mm self-adhesive nylon cable tie mounting pad was adhered approximately to the center of the upper edge of the fiberglass door of the feeding system (Figure 1B-1). A 6.3 mm × 50.8 mm zinc-plated steel eye bolt was fixed approximately perpendicular to the mounting pad in the right lateral wood panel of each feed bunk (Figure 1B-5) and the sensor bracket was loosely attached with a nylon cable tie. A braided nylon string (Figure 1B-2) was used to tie the sensor bracket (Figure 1B-4) from the elastic band (Figure 1B-3) to the cable tie mounting pad. When tying the sensor system, the tension was set to be lower than the tension of the spring that closes the fiber glass door in the feeding system to prevent interference with closure of the door. Sensors were checked twice daily to correct visible sag due to loss of tension and ensure the sensors stayed in position. Sensors were programmed to record position in all 3 axes every 30 s to allow calculation of tilt angle.

All experimental procedures were approved by The Pennsylvania State University Institutional Animal Care and Use Committee (PROTO201900784). Twenty-four multiparous and 24 primiparous cows were arranged in a crossover design in a concurrent trial investigating the interaction between genetic polymorphisms and dietary acetate supplementation (data not shown). Cows were housed in a freestall barn at the Pennsylvania State University Dairy Production Research and Teaching Center equipped with a Calan Broadbent Feeding System. Cows were milked twice daily at 0600 and 1800 h and fed once daily at approximately 0800 h. Cows were locked out of the feed alleyway at 0500 h daily to allow for cleaning and weighing orts. Cows were also restricted from accessing the bunks upon return from morning milking until all bunks had feed to prevent a bias in the data caused by anticipatory behavior while feed was being delivered to the bunks. Experimental periods were 14 d with a 10-d washout period in between, and the sensors were set up during the last 7 d of each experimental period and installed while cows were out of the pen for milking. Recording was programmed to start before feeding on the first day of deployment and terminated after retrieval on the last day of each period. The sensors’ recording missions were set up using the same computer in all instances to synchronize timestamps of each recorded observation (Dell Latitude E5570 with Windows 10 Education ver. 21H1 with United States regional settings and UTC-05:00 as the local time zone). Two sensors malfunctioned during one of the observation periods, resulting in complete data loss (likely battery failure). A total of 11 d of data were lost because of sensors becoming detached from the Calan feeding door. The sensors were not accessible to the cows, and malfunctions were not caused by interference of the cows.

Data were downloaded from each sensor and plotted with HOBOware ver. 3.7.20 (Onset Computer Corp.) to visually assess data viability. Tilt angle at each axis was automatically calculated [tilt angle = 180° – ArcCos (acceleration in *g*)]. Sensor setup was designed to best capture the movement of the door on the Z-axis, thus the tilt at the Z-axis was exported. It is important to note that the sensor has the capability of measuring tilt angle on all 3 axes, thus the setup can be adapted to capture the movement of the door in other axes if needed. The data from each sensor were then divided into experimental days as the 24-h period between feed delivery (i.e., approximately between 0500 h of adjacent days) using the subset function in R ver. 4.0.1 (https://www.r-project.org/).

A minimum Z-tilt angle threshold was used to determine when the door was opened or closed. By design, when the door was closed, the sensor read ~0° Z-tilt angle and when fully open, it read >60° Z-tilt angle (Figure 1A). A threshold of 30° was used to define an open door to account for when the sensor sagged during the observation period. Using a threshold of 30° did not exclude events in which the cows were eating in the bunk, as the feed was inaccessible in this position. Furthermore, the first 3 bins of the count distribution of all raw Z-tilt angles >0° are most likely Z-tilt observations of a closed door with a sensor contraption that has sagged or a sensor that was not near 0° in its resting state (Figure 1B). When the first 3 bins were ignored, 30° was below the 2.5th percentile of the distribution. Thus, when the sensor read a Z-tilt angle <30°, the measurement was coded as “0” to represent that the door was closed; if the sensor read ≥30°, it was coded as “1” to represent that the door is open in a new column created with the “mutate” function in the dplyr package in R (Hadley et al., 2020). The running series of consecutive annotations was then calculated with the “streak_run” function in the runner package in R (Kaledkowski, 2020) to calculate the between-feeding intervals. An annotated sample of the R code used to process the data is available at the “Door Sensor Feeding Behavior” public GitHub repository (https://github.com/cesarimg/HarvatineLab.git) or upon request to the corresponding author.

The sensor system was validated by comparison with visual feeding behavior monitoring. Briefly, the sensors were set up as described above in 48 Calan Broadbent doors, and recording was programmed to record an observation in all 3 axes once per second to allow calculation of tilt angle. Tilt angle on the Z-axis was used as above to determine whether the door was open or closed. Three trained observers manually recorded (in Google Docs) whether the doors were closed or open due to feeding activity for 6 h starting immediately after morning feeding (0800 h) for 1 d. Average recording frequency for the visual observations was 1 visual observation every 2 min and 50 s. A time stamp was automatically recorded with entry of each observation, and data from the sensor and visual observations were aligned based on the time stamps. The mode of the 5 previous and following sensor observations was used to correct for any disturbances to the cow’s behavior from the observer’s presence and movement in the barn. Cohen’s κ was calculated using the frequency of open-close determination by both methods to test the agreement between them, as described by Watson and Petrie (2010; Figure 1C). The calculated κ value was 0.92 ± 0.014 (estimate ± 95% CI), suggesting very high agreement between methods. The positive predictive value of the sensors, which is the probability of the measurement of an open state by the sensor being correct, was 98%. The negative predictive value of the sensors, which is the probability of the measurement of a closed state being correct, was 97%. Therefore, the sensor system accurately determines the state of the door compared with visual observations. It is important to note that the system was validated for observation of the presence of a cow in the feeding gate, not that the cow was eating. Future work should further validate time at the feed bunk with eating.

To characterize meals in feeding behavior analysis, it is necessary to determine a minimum intermeal interval, also known as the meal criterion, which is the minimum length required between 2 feeding bouts to be considered separate events. Calculating this criterion is important because it directly affects characterization of meals. The minimum intermeal interval differs between feeding systems and was determined by fitting multiple normal distributions to the log-transformed intervals, as described by Tolkamp et al. (2011). The normal distributions were fit using the continuous fit mode in the distribution module of JMP Pro 14.3.0 (SAS Institute Inc.). Two and 3 normal distribution models were fit, and the best fit was determined by the lowest value of maximum likelihood and corrected Akaike’s and Bayesian information criteria (AICc and BIC, respectively; Schwarz, 1978; Sugiura, 1978). The minimum intermeal interval was defined as the point at which the Gaussian probability density functions intersect (***C***), which was calculated using a derivative of the quadratic formula as in Anderson et al. (2009), using the distribution parameters if *μ*_*1*_ *< μ*_2_ and *σ*_*1*_ ≠ *σ*_*2*_:

[1] $$C=\frac{\mu_{2}\sigma_{1}^{2}-\sigma_{2}\left(\mu_{1}\sigma_{2}+\sigma_{1}\sqrt{{\left(\mu_{1}-\mu_{2}\right)}^{2}+2\left(\sigma_{1}^{2}-\sigma_{2}^{2}\right)\log\left(\sigma_{1}/\sigma_{2}\right)}\right)}{\sigma_{1}^{2}-\sigma_{2}^{2}},$$

where *μ*_1_ and *σ*_1_ and *μ*_2_ and *σ*_2_ are the mean (*μ*) and standard deviation (*σ*) of the utmost right distributions in the data. Meal length was calculated as the summation of intervals between events where the door was closed for more than the minimum intermeal interval. Meal frequency was calculated by cow within day by calculating the running series of meals within days as above. Distribution analysis for meal length and frequency was done in JMP PRO 15.0.0 (SAS Institute Inc.).

The sensors were not observed to interfere with normal operation of the door. The design is minimally intrusive and does not permanently modify the door system. The sensors did sag slightly over time with approximately 5% of the data points (approximately 83,000 observations from a total of ~1.5 million observations) resulting in Z-tilt between 0° and 30°, but sufficient dynamics between open and closed remained to allow analysis. It is recommended that the sensors be monitored during experiments and tightened if needed.

Defining the minimum intermeal interval is essential to determining meals. There was a considerable number of “door closed” intervals less than 2 min in the raw data (10,974 intervals of a total 17,468 intermeal intervals or ~63% of observed intervals). These were omitted when fitting the normal distributions to define a minimal intermeal interval because it has been characterized to be at least 8 min in other systems (Dado and Allen, 1993; Niu et al., 2017). Additionally, tilt angle was only observed every 30 s, and some of the intervals are expected to be less than 30 s. Adequately characterizing the distribution of short intermeal intervals would require a smaller recording frequency than that of the shortest expected interval (Tolkamp et al., 1998). The sensors allow for higher recording frequencies (up to recordings every second), but have limited memory so data must be downloaded more often. Fill time of the sensor’s memory is a function of how many axes are being logged and the recording frequency (number of samples/s), and can be estimated with the following equation:

[2] $$\;\text{Fill}\;\text{time}\;(\text{s})=\frac{\;\text{Memory}\;\text{size}\;-\;\text{Output}\;\text{header}\;\text{size}\;}{\;\text{No}.\;\text{of}\;\text{samples}/\text{s}\times \;\text{No}.\;\text{of}\;\text{axes}\;\text{enabled}\;}\text{,}\;$$

where memory size is approximately 65 kB and the typical output header size is 500 kB (Onset Computer Corp., 2013). Thus, increasing the recording frequency of the sensors should be weighted by the amount of time desired for observation. The selected 30-s resolution allowed collection of 7.5 d of data and was 1.5% of the estimated minimum intermeal intervals, indicating sufficient resolution for characterization of meal bouts.

In characterizing the minimum intermeal interval, the distribution that best fit the log-transformed intervals was a mixture of 3 Gaussian distributions (−2LogLikelihood = 20,729.3, AICc = 20,745.3, and BIC = 20,799.5; Figure 2B). Using the intersection of the last 2 distributions (*μ*_1_ = 6.34, *σ*_1_ = 0.61, *μ*_2_ = 8.77, *σ*_2_ = 0.63), the minimum intermeal interval was calculated to be approximately 31.3 min, which is similar to that reported for cows in free housing [DeVries et al. (2003) reported 27.7 min, Huzzey et al. (2005) reported 33.3 min, DeVries et al. (2007) reported 26 min, Tolkamp et al. (2011) reported 30 min, and Moore and DeVries (2020) reported an average of 29.7 min]. The interval in group-housed cows appears to be substantially different from that in tie stalls, as Dado and Allen (1993) reported a minimum intermeal interval of 7.5 min for electronic observations based on a chew halter and variation in a hanging feed tub, and Niu et al. (2017) reported a minimum meal interval of 8 min using feed tubs suspended from load monitors in tie stall–housed cows. The minimum intermeal interval in the current project was consistent when considering the second-best fitting distribution, a mixture of 2 Gaussian distributions (−2LogLikelihood = 21,160.5, AICc = 21,170.5, and BIC = 21,204.4; Figure 2B), which resulted in a minimum intermeal interval of 27.4 min (*μ*_1_ = 5.97, *σ*_1_ = 0.69, *μ*_2_ = 8.75, *σ*_2_ = 0.65). The distribution clusters are most likely characterizing the inter- and intrameal intervals and pauses taken from eating to drink water (Tolkamp et al., 2011).

Using the minimum intermeal interval of 31.3 min, the computed meal length averaged 37.3 ± 28 min with 10th and 90th percentiles of 11 and 78 min/meal, respectively. Meal frequency averaged 7.3 ± 1.4 meals/d with 10th and 90th percentiles of 6 and 9 meals/d, respectively (mean ± SD for both; Figure 3). This agrees with DeVries et al. (2007) and Moore and DeVries (2020) for cows housed in similar conditions. Feeding behavior analysis also commonly uses a defined minimum meal size or length. Sensitivity analysis has shown that the calculated DMI accounted for by feeding behavior systems decreases as the minimum meal size and length criteria increase (Heinrichs and Conrad, 1987). To the best of our knowledge, there is no published objective methodology to determine minimum meal size or length. Authors usually rely on subjective predetermined thresholds such as a minimum meal size (as-fed basis) of 1 kg and a minimum meal length of 30 s (see, for example, Harvatine and Allen, 2006). Minimum meal length, the secondary criterion for the described system, has not been reported to our knowledge. The frequency distribution did not appear to include multiple clear populations precluding use of modeling multiple distributions similar to the minimum intermeal interval. In total, 0.42% of meals were 30 s or less, 0.79% were less than 60 s, and 1.61% were 120 s or less. Meals that had a length of 30 s were excluded from the final analysis (total of 15 out of 3,537 meals) because there was no way to confirm whether the cow was in the bunk for the entirety of the 30 s, effectively making our minimum meal length criteria of >30 s. Future work should be conducted to determine a physiologically relevant minimum meal length.

In conclusion, a data-logging 3-axis accelerometer is adequate to monitor feeding behavior in Calan Broadbent Feeding System similar to that previously validated using a change-of-state sensor. When validated against manual observations, the sensor system was in very high agreement with manual observations. The minimum intermeal interval of ~30 min agrees with previous reports in freestall-housed lactating dairy cows and allows for reasonable estimations of meal length and frequency. The design uses commonly available components and minimal setup and labor and should serve as a blueprint that future users can modify or improve to suit other specific constraints. This will allow collection of important information on the effect of treatments on feeding behavior in experiments using the Calan Broadbent Feeding System.

## Figures and Tables

**Figure 1.**
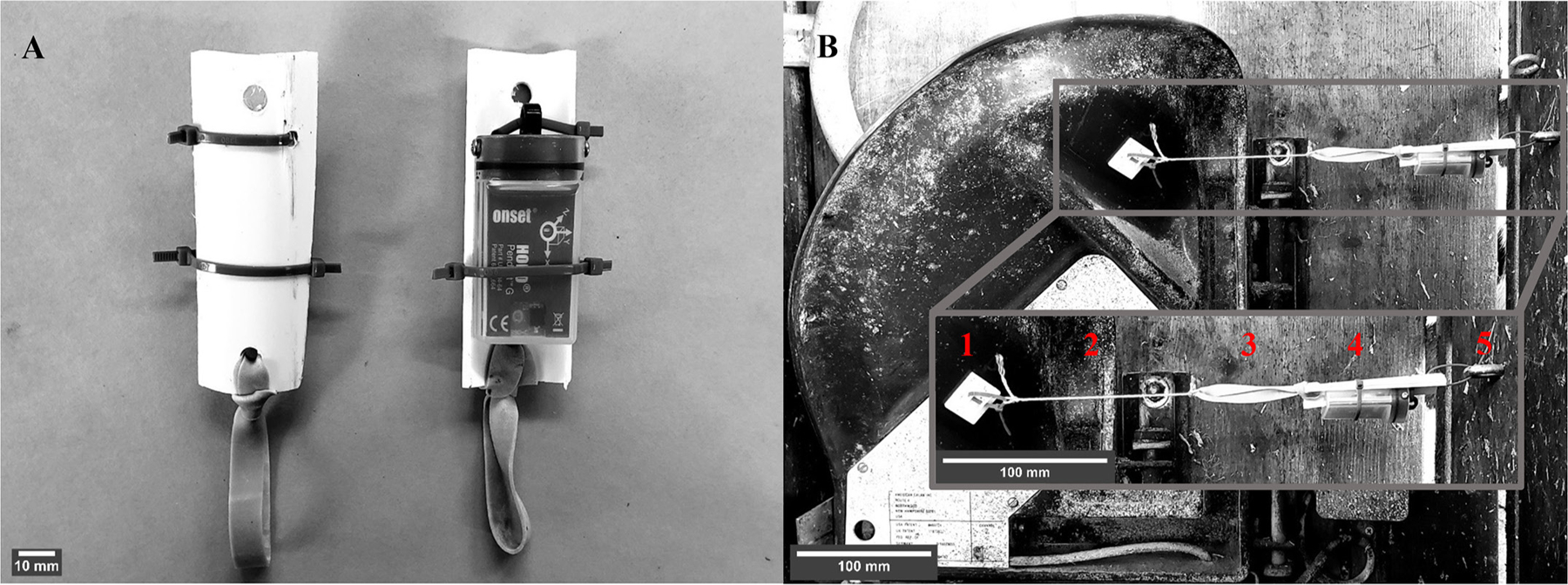
Mounting a data-logging accelerometer to a Calan Broadbent door (American Calan). (A) A HOBO Pendant G sensor (Onset Computer Corp.) is attached with nylon cable ties to a strip of polyvinyl chloride (PVC) pipe with an elastic band at one end. (B) The sensor is strung between the door feeding divider. It is attached to the door using a self-adhesive nylon cable tie mounting pad (1), and a braided nylon string (2) is fastened to the rubber band (3) attached to the sensor support (4). The sensor support is then loosely attached with a nylon zip tie to a zinc-plated steel eye bolt (5) in the wood wall of the feed bunk. Scale bars were added using ImageJ 1.53e (https://imagej.nih.gov/ij/).

**Figure 2.**
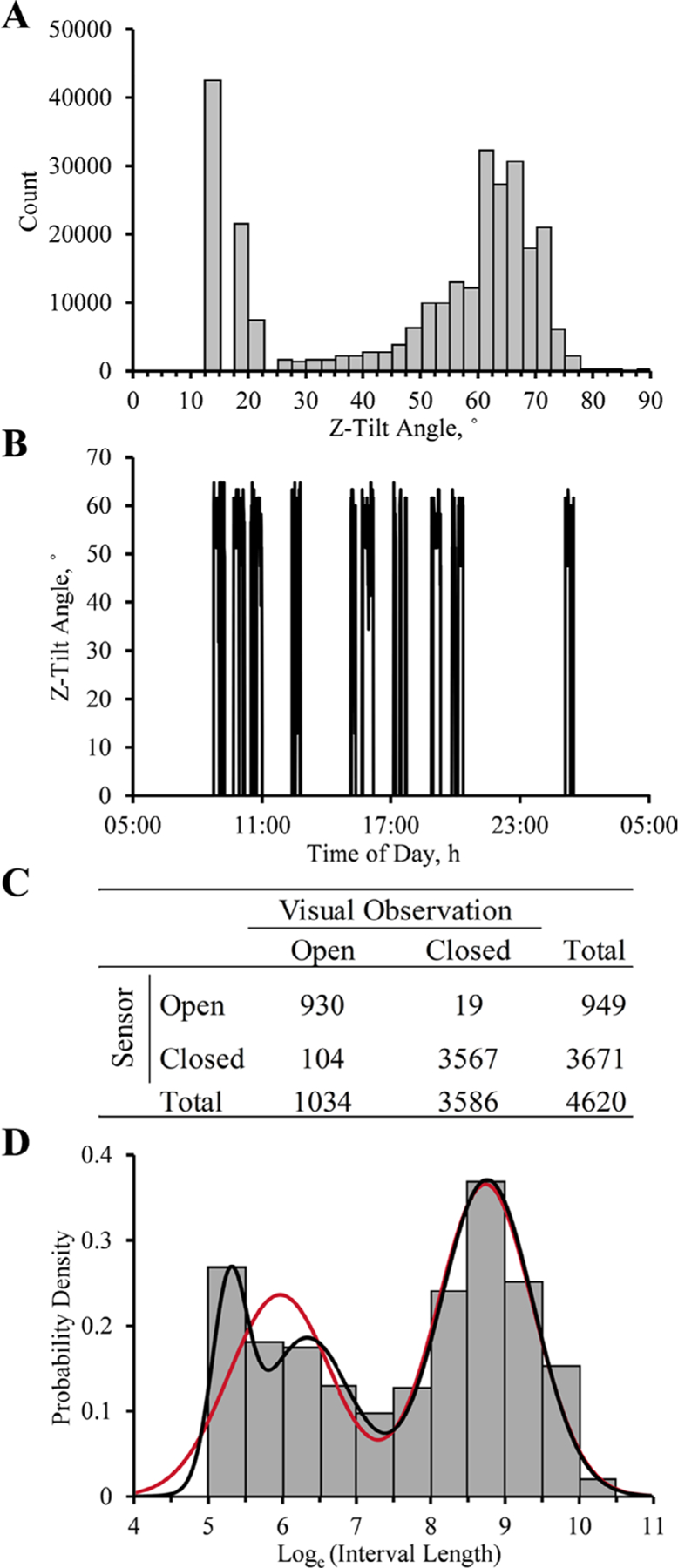
Feeding behavior analysis using a data-logging 3-axis accelerometer attached to a Calan Broadbent door (American Calan). (A) Count distribution of nonzero Z-tilt angles collected every 30 s throughout the observation period in the experiment. Zero values were omitted to adjust the scale of y-axis for other observed angles (0° count ~1.1 million observations). (B) Raw Z-tilt angle collected every 30 s from a representative cow for 24 h after feed delivery. (C) Contingency table of frequencies of open-closed door determination using visual observations or a 3-axis accelerometer. (D) Frequency distribution of the log-transformed between-feeding intervals divided by bin width (0.5 log_*e*_-units; gray bars) and the fit of a probability density function containing a mixture of 2 (red line) or 3 (black line) Gaussian distributions.

**Figure 3.**
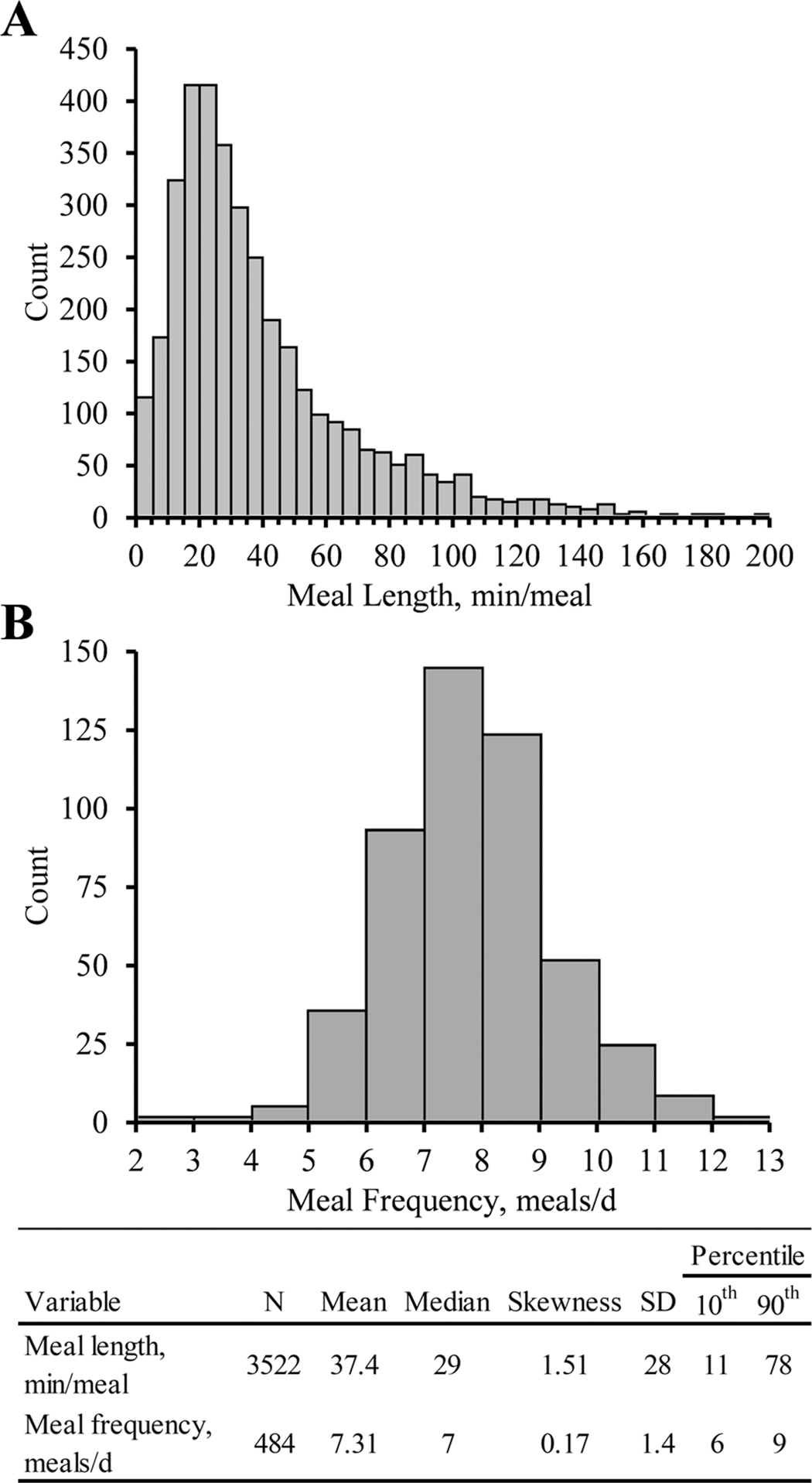
Estimated meal frequency and length from data collected using a data-logging accelerometer attached to a Calan Broadbent door (American Calan). (A) Count distribution of meal frequency (meals/d). (B) Count distribution of meal length (min/meal). Select distribution statistics are shown in a table at the bottom for both meal characteristics.

## Highlights

- Accelerometers were used as an alternate method to monitor feeding behavior in Calan gates.
- There was a very high level of agreement between the accelerometers and visual observations of feeding.
- Accelerometers appear to be a valid method to measure feeding behavior in Calan gates

## References

[R1] Allen MS 2014. Drives and limits to feed intake in ruminants. Anim. Prod. Sci 54:1513–1524. 10.1071/AN14478.

[R2] Anderson G, Ge Y, and Leo TW 2009. Distributional overlap: Simple, multivariate, parametric, and nonparametric tests for alienation, convergence, and general distributional difference issues. Econom. Rev 29:247–275. 10.1080/07474930903451532

[R3] Bach A, Iglesias C, and Busto I 2004. Technical note: A computerized system for monitoring feeding behavior and individual feed intake of dairy cattle. J. Dairy Sci 87:4207–4209. 10.3168/jds.S0022-0302(04)73565-1 15545384

[R4] Chapinal N, Veira DM, Weary DM, and Von Keyserlingk MAG 2007. Technical note: Validation of a system for monitoring individual feeding and drinking behavior and intake in group-housed cattle. J. Dairy Sci 90:5732–5736. 10.3168/jds.2007-0331. 18024766

[R5] Costa JHC, Cantor MC, and Neave HW 2021. Symposium review: Precision technologies for dairy calves and management applications. J. Dairy Sci 104:1203–1219. 10.3168/jds.2019-17885. 32713704

[R6] Dado RG, and Allen MS 1993. Continuous computer acquisition of feed and water intakes, chewing, reticular motility, and ruminal pH of cattle. J. Dairy Sci 76:1589–1600. 10.3168/jds.S0022-0302(93)77492-5.

[R7] DeVries TJ, Beauchemin KA, and Von Keyserlingk MAG 2007. Dietary forage concentration affects the feed sorting behavior of lactating dairy cows. J. Dairy Sci 90:5572–5579. 10.3168/jds.2007-0370. 18024749

[R8] DeVries TJ, Von Keyserlingk MAG, Weary DM, and Beauchemin KA 2003. Measuring the feeding behavior of lactating dairy cows in early to peak lactation. J. Dairy Sci 86:3354–3361. 10.3168/jds.S0022-0302(03)73938-1. 14594255

[R9] Hadley W, François R, Henry L, and Müller K 2020. dplyr: A Grammar of Data Manipulation. https://cran.r-project.org/web/packages/dplyr/index.html.

[R10] Harvatine KJ, and Allen MS 2006. Effects of fatty acid supplements on feed intake, and feeding and chewing behavior of lactating dairy cows. J. Dairy Sci 89:1104–1112. 10.3168/jds.S0022-0302(06)72178-6. 16507707

[R11] Heinrichs AJ, and Conrad HR 1987. Measuring feed intake patterns and meal size of lactating dairy cows. J. Dairy Sci 70:705–711. 10.3168/jds.S0022-0302(87)80061-9. 3584609

[R12] Huzzey JM, Von Keyserlingk MAG, and Weary DM 2005. Changes in feeding, drinking, and standing behavior of dairy cows during the transition period. J. Dairy Sci 88:2454–2461. 10.3168/jds.S0022-0302(05)72923-4. 15956308

[R13] Johnston C, and DeVries TJ 2018. Short communication: Associations of feeding behavior and milk production in dairy cows. J. Dairy Sci 101:3367–3373. 10.3168/jds.2017-13743. 29397173

[R14] Kaledkowski D 2020. runner: Running Operations for Vectors. https://cran.r-project.org/web/packages/runner/index.html.

[R15] Krawczel PD, Klaiber LM, Thibeau SS, and Dann HM 2012. Technical note: Data loggers are a valid method for assessing the feeding behavior of dairy cows using the Calan Broadbent Feeding System. J. Dairy Sci 95:4452–4456. 10.3168/jds.2011-4999. 22818459

[R16] Ledgerwood DN, Winckler C, and Tucker CB 2010. Evaluation of data loggers, sampling intervals, and editing techniques for measuring the lying behavior of dairy cattle. J. Dairy Sci 93:5129–5139. 10.3168/jds.2009-2945. 20965328

[R17] Mendes EDM, Carstens GE, Tedeschi LO, Pinchak WE, and Friend TH 2011. Validation of a system for monitoring feeding behavior in beef cattle. J. Anim. Sci 89:2904–2910. 10.2527/jas.2010-3489. 21512115

[R18] Moore SM, and DeVries TJ 2020. Effect of diet-induced negative energy balance on the feeding behavior of dairy cows. J. Dairy Sci 103:7288–7301. 10.3168/jds.2019-17705. 32475669

[R19] Niu M, Ying Y, Bartell PA, and Harvatine KJ 2014. The effects of feeding time on milk production, total-tract digestibility, and daily rhythms of feeding behavior and plasma metabolites and hormones in dairy cows. J. Dairy Sci 97:7764–7776. 10.3168/jds.2014-8261. 25306274

[R20] Niu M, Ying Y, Bartell PA, and Harvatine KJ 2017. The effects of feeding rations that differ in fiber and fermentable starch within a day on milk production and the daily rhythm of feed intake and plasma hormones and metabolites in dairy cows. J. Dairy Sci 100:187–198. 10.3168/jds.2016-11129. 27837979

[R21] Onset Computer Corp. 2013. HOBO ® Pendant ® G Data Logger (UA-004–64) White Paper.

[R22] Overton MW, Sischo WM, Temple GD, and Moore DA 2002. Using time-lapse video photography to assess dairy cattle lying behavior in a freestall barn. J. Dairy Sci 85:2407–2413. 10.3168/jds.S0022-0302(02)74323-3. 12362476

[R23] Salfer IJ, and Harvatine KJ 2020. Night-restricted feeding of dairy cows modifies daily rhythms of feed intake, milk synthesis and plasma metabolites compared with day-restricted feeding. Br. J. Nutr 123:849–858. 10.1017/S0007114520000057. 31910910 PMC7154010

[R24] Schwarz G 1978. Estimating the dimension of a model. Ann. Stat 6:461–464. 10.1214/aos/1176344136.

[R25] Sugiura N 1978. Further analysis of the data by Akaike’s information criterion and the finite corrections. Commun. Stat. Theory Methods 7:13–26. 10.1080/03610927808827599.

[R26] Tolkamp BJ, Allcroft DJ, Austin EJ, Nielsen BL, and Kyriazakis I 1998. Satiety splits feeding behaviour into bouts. J. Theor. Biol 194:235–250. 10.1006/jtbi.1998.0759. 9778436

[R27] Tolkamp BJ, Allcroft DJ, Barrio JP, Bley TAG, Howie JA, Jacobsen TB, Morgan CA, Schweitzer DPN, Wilkinson S, Yeates MP, and Kyriazakis I 2011. The temporal structure of feeding behavior. Am. J. Physiol. Regul. Integr. Comp. Physiol 301:R378–R393. 10.1152/ajpregu.00661.2010. 21525177

[R28] Vasilatos R, and Wangsness PJ 1980. Feeding behavior of lactating dairy cows as measured by time-lapse photography. J. Dairy Sci 63:412–416. 10.3168/jds.S0022-0302(80)82948-1. 7372905

[R29] Watson PF, and Petrie A 2010. Method agreement analysis: A review of correct methodology. Theriogenology 73:1167–1179. 10.1016/j.theriogenology.2010.01.003. 20138353

